# YAAM: Yeast Amino Acid Modifications Database

**DOI:** 10.1093/database/bax099

**Published:** 2018-01-09

**Authors:** Leonardo Ledesma, Eduardo Sandoval, Uriel Cruz-Martínez, Ana María Escalante, Selene Mejía, Paola Moreno-Álvarez, Emiliano Ávila, Erik García, Gerardo Coello, Francisco Torres-Quiroz

**Affiliations:** 1 Unidad de Cómputo, Instituto de Fisiología Celular, Universidad Nacional Autónoma de México, México, Ciudad de México 04510, México; 2 División de Ciencia Básica, Departamento de Bioquímica y Biología Estructural, Instituto de Fisiología Celular, Universidad Nacional Autónoma de México, México, Ciudad de México 04510, México; 3 Coordinación de Difusión y Divulgación, Instituto de Fisiología Celular, Universidad Nacional Autónoma de México, México, Ciudad de México 04510, México

## Abstract

Proteins are dynamic molecules that regulate a myriad of cellular functions; these functions may be regulated by protein post-translational modifications (PTMs) that mediate the activity, localization and interaction partners of proteins. Thus, understanding the meaning of a single PTM or the combination of several of them is essential to unravel the mechanisms of protein regulation. Yeast Amino Acid Modification (YAAM) (http://yaam.ifc.unam.mx) is a comprehensive database that contains information from 121 921 residues of proteins, which are post-translationally modified in the yeast model *Saccharomyces cerevisiae*. All the PTMs contained in YAAM have been confirmed experimentally. YAAM database maps PTM residues in a 3D canvas for 680 proteins with a known 3D structure. The structure can be visualized and manipulated using the most common web browsers without the need for any additional plugin. The aim of our database is to retrieve and organize data about the location of modified amino acids providing information in a concise but comprehensive and user-friendly way, enabling users to find relevant information on PTMs. Given that PTMs influence almost all aspects of the biology of both healthy and diseased cells, identifying and understanding PTMs is critical in the study of molecular and cell biology. YAAM allows users to perform multiple searches, up to three modifications at the same residue, giving the possibility to explore possible regulatory mechanism for some proteins. Using YAAM search engine, we found three different PTMs of lysine residues involved in protein translation. This suggests an important regulatory mechanism for protein translation that needs to be further studied.

**Database URL**: http://yaam.ifc.unam.mx/

## Introduction

Over time, living organisms have evolved through different regulation levels to control cell behaviour, not merely to survive and grow, but also to trigger proper responses to external stimuli. One of these regulatory levels is the Post-Translational Modifications (PTMs), covalent modifications of a single or multiple amino acids in a protein. These versatile PTMs have become a broad field of study due to the chronological variability of their action that can go from seconds—as in the case of phosphorylation—to slower responses—as in glycosylation (1).

PTMs target specific sites in proteins hence having many effects on protein behaviour; a single PTM is able to change the location of a protein and its interaction with DNA, lipids and other proteins; PTMs could also affect function by hiding or exposing the catalytic site or changing substrate binding affinity. It is believed that these functions can be driven by a ‘PTM code’ (1, 2), from this perspective a specific combination of PTMs can trigger or inhibit a series of downstream events ultimately leading to a fully integrated signal in the cell (3).

The main sources of information on PTMs come from mass spectrometry (MS) studies; these different MS methods used to obtain PTMs data, have different degrees of sensitivity and have generated results with various degrees of robustness (4–6). The emergence of these MS studies have led to an increase in the amount of data available in the literature, which in turn has created the need to collect and make this information easier to manage and analyse. Furthermore, this has allowed the development of new models and approaches for a better understanding of the mechanism of action of PTMs and, it has improved the interpretation of the relationships among these regulatory modifications (7).

In order to address this need, several PTM databases have been created (8–12); most of them are focused on different model organisms and include data of an important number of PTMs. Besides that, these databases gather large amount of data, which is difficult to visualize and understand if one aims to analyse all the sites for a specific modification from a determinate organism (9, 11, 12). Additionally, some of these databases are outdated (9, 12) (Supplementary Table S1), what makes a complicate task to obtain reliable information from them.

Since no single database is devoted entirely to budding yeast’s PTMs, and considering that *S. cerevisiae* is an important model organism, we dedicated our efforts to create a database focused exclusively in the PTMs that occur in this budding yeast. By creating this database, we collected for the first time the PTMs that are meaningful for this model organism (121 921 PTMs), and we included, as well, additional structural and functional significant data for budding yeast, such as active sites, metal-binding and calcium-binding sites (information from 3089 residues). Although there are databases that include data addressing PTMs in yeast, none of them comprise some characteristics that we consider of major importance for yeast physiology. Furthermore, our database enables easy access to systematic information, within a user-friendly environment that shows not only the residues that are being modified in a linear sequence, but also, where available, the location of these amino acid in a 3D map of the protein. Our effort is aimed to give a better understanding of PTMs spatial location by offering users synthesized data. Additionally, our database seek to integrate this data with functional insights providing information on protein sub-cellular localization and the surroundings of modified residues that are important to each protein.

Although the simple location of PTMs in a protein sequence is not enough to infer the regulatory significance of those modifications, we consider that this kind of data visualization will help expert users to elucidate protein regulation mechanisms analysing the PTM context where a modification occurs. 

## Materials and methods

### Data compilation for YAAM database

The systematic names, standard names, aliases, descriptions, protein sequences and Gene Onthology (GO)-Slim terms in Yeast Amino Acid Modification (YAAM) were obtained from the *Saccharomyces* Genome Database (13). The active site, ions binding sites, disulfide-forming residues, glycosylation and lipidation are from UniProt (14). The most part of the phosphorylation data is from Phosphogrid (15), some phosphorylation, acetylation and ubiquitination sites are from PTMfunc (4). The remaining data on PTM sites were manually curated from high-throughput MS studies (5, 16–22) or obtained from literature manually curated. The protein localization was based on the GO-Slim terms; the cellular component classification was compiled and the annotations were reduced, accordingly to yeast physiology, to 16 categories that are easy to find using a fluorescent fusion protein under a fluorescence microscope. For the proteins with a classification out of the 16 categories, we manually compared the localization using published information on the protein and comparing localization databases (23–25). All the parsers for the data compilation were performed using Perl (version 5.10.1) or/and Python (version 2.7.10).

### 3D structure visualization

The 3D structures were generated with PDB files from the RCSB Protein Data Bank (26). There are 3109 proteins associated with *Saccharomyces cerevisiae* in the RCSB Protein Data Bank, after selecting the files with only one molecule or the minor number of molecules per PDB file we obtained 709 structures. The sequences from the PDB file with the protein sequence from our database were aligned; the PDB files with mismatches were depurated and we obtained 680 PDB files associated with proteins. Given that the format in PDB files for the protein name is not uniform and the information in the webpage and the downloaded files was not always consistent, the 680 proteins with at least one crystal structure described were manually curated in order to show the longest 3D structure with the maximum number of modified residues.

### Analysis for coincidences of modified residues

Using YAAM we obtained a list of proteins acetylated, ubiquitilated and succinylated at the same residue. We performed a Gene Ontology Enrichment analysis of the selected proteins using YeastMine (27). As background for the analysis, we used all the proteins that are acetylated, ubiquitilated or succinylated. For the test correction, we used the Holm–Bonferroni method with a maximum *P*-value of 0.05.

## Database architecture and web interface

The conceptual and logical model of YAAM was created using Edraw Max 7.0 (https://www.edrawsoft.com/EDrawMax.php). We built and configured YAAM based on the open source platform LAMP (Linux-Apache-MySQL-PHP). The database is deployed on an Apache server 2.0 (http://www.apache.org). The physical model was implemented with MySQL 5.1.73, and it consists of 20 tables with 164 242 records. The server-side programming was implemented using PHP version 5.3.3. The web interface was created using JavaScript, HTML 5 and CSS 3.

The YAAM interface is easy-to-use, allowing an easy search and visualization of results. YAAM has five main types of pages: *HOME, YAAM SEARCH, ADD YAAM, TUTORIAL, LAST UPDATE and PROTEIN DETAIL*.

**Figure 1.**
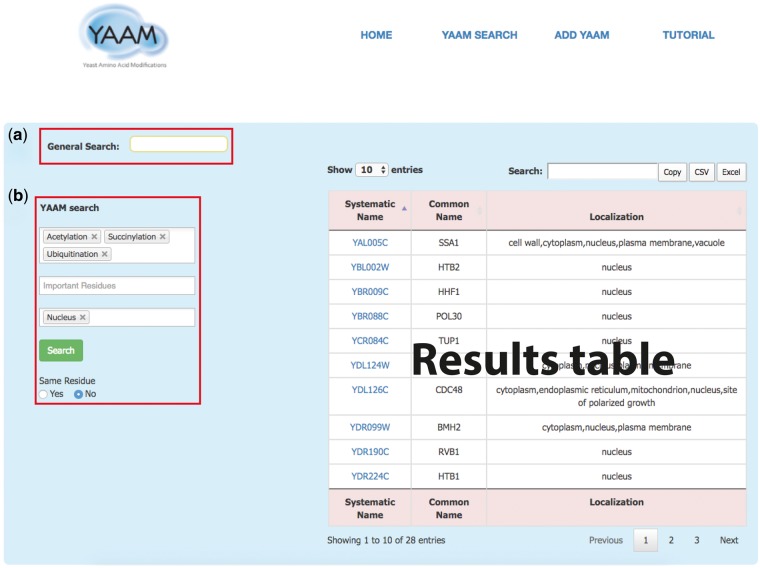
The *YAAM search* page enables the user to perform two types of search; (**a**) a *General Search* (upper red square) where the user could search by words, and (**b**) the *YAAM search* (lower red square) where is possible to search up to three different options of PTMs, active site or ion binding site and up to three options of localization sites. Additionally, the *YAAM search* can be restricted to search for modifications in the same amino acid residue. In the example, we show a *YAAM search* for acetylated, succinylated and ubiquitinated proteins in any residue with nuclear localization.

*YAAM SEARCH*: in this page YAAM allows making two types queries: a *General search* and a *PTM search* (*YAAM search*) (Figure 1). In the general search, proteins can be found by name, systematic name, alias, key word, Uniprot/Swiss-prot ID, NCBI gene ID and NCBI RefSeq protein version ID. In the *YAAM search* field, one or more modifications of interest and important residues can be preselected from a dropdown list. The sub-cellular localization can also be selected from a dropdown list. The search can also be narrowed by coincidences in the same residue. In the case of *YAAM search*, the Boolean logical operator ‘AND’ is applied to the database to create a subset of search results. This search strategy restricts results, displaying only the information with the specified criteria of the *YAAM search*.

In both type of search queries, results are displayed in a list containing the systematic name, the common name and the sub-cellular localization of the protein. The tables were formatted using the open source plug-in for the jQuery Javascript library DataTables (http://datatables.net/). At the top of the table there is a box where the user can filter information from any of the three columns. Furthermore, all database information is available to download as .txt at the bottom of each page and data sets generated in YAAM are available for download as .csv or .xls files, or copying. Finally, the generated table could be analysed in YeastMine for enrichment analysis, but only for lists with <400 entries.

**Figure 2.**
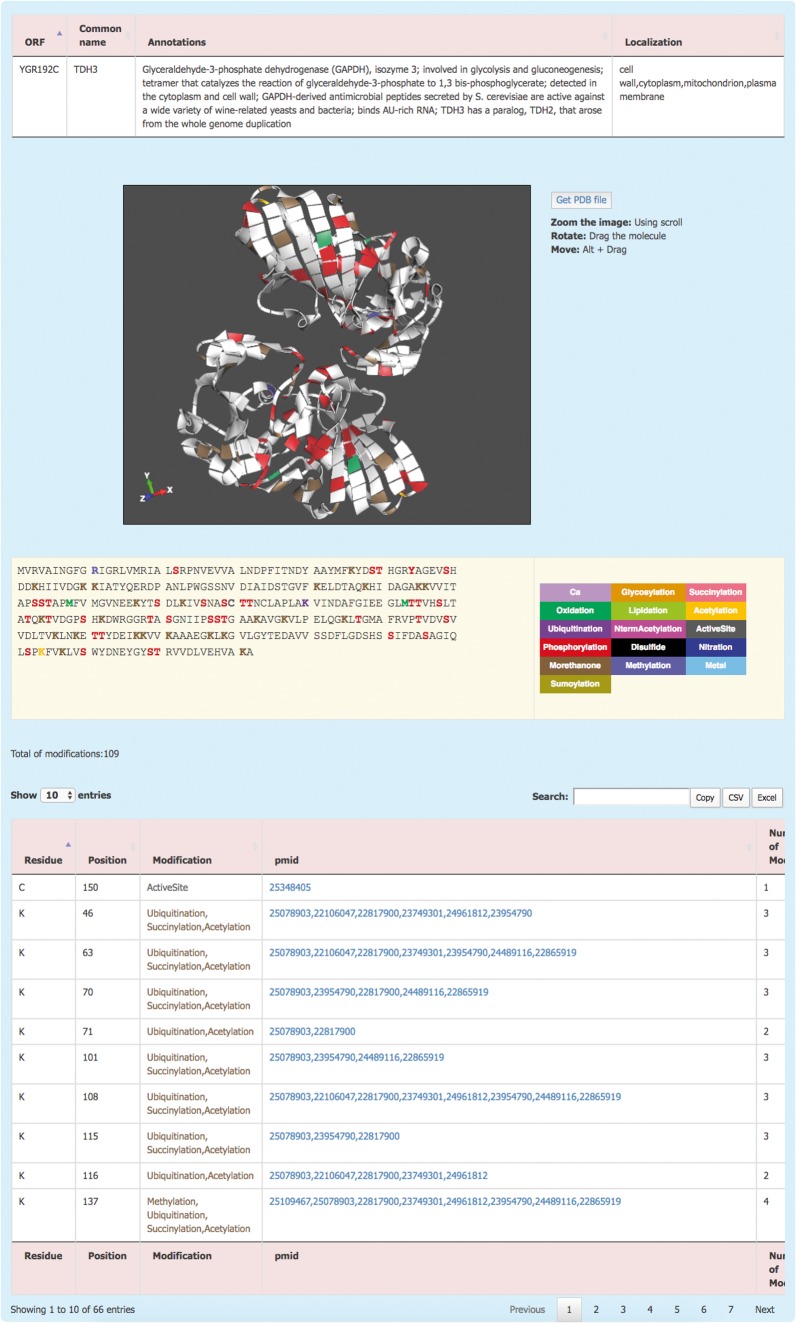
The YAAM Protein description page shows general information about the protein, the modified residues mapped in a 3D structure (when available), the linear sequence with the modified residues depicted with a colour code, the total number of modifications and, a table with the description of each one.

The information about each protein is displayed by clicking on the systematic name (Figure 2). This redirects the user to the *Protein details* page where the PTM information of the protein is displayed. The *Protein details* page shows a brief description of the protein and its sub-cellular localization. For the proteins with an available 3D structure, YAAM displays a visualization window with a 3D model of the protein where each modification is mapped. The molecule can be zoomed, rotated or moved by using the scroll, dragging the molecule or clicking Alt + dragging the molecule, respectively. The .pdb file of the protein is available for download by clicking the ‘Get PDB file’ button. The 3D structures are visualized with the ChemDoodle Web Components library (http://web.chemdoodle.com/) an open-source javascript chemical graphic library that requires no plugins and works in the most used browsers as it is based on WebGL and HTML5.

PTM amino acids are visualized in a linear sequence with the total number of PTMs reported for the protein; each residue subject to modifications or considered important for the protein activity is marked according to a colour code displayed on the right side of the sequence. In order to have the exact number of PTMs, the total number of modifications is displayed at the bottom of the sequence. Additionally, a list with all the information is showed; this information include the residue, position, type of modification, the PubMed Unique Identifier where PTM was reported and finally the total number of modifications for each residue. At the top of the table, there is a box where the user can filter information from any of the five columns. Furthermore, the information from this table is available for copying or downloading as a .csv or .xls files.

YAAM is a dynamical, manually curated database that could be updated not only by its creators but also by users who are encouraged to contribute by sending their new findings. The *ADD A YAAM* page offers a submission form that allows users to send experimentally validated information on PTMs. This option enables the scientific community to submit data in order to keep YAAM updated by means of users’ contribution. Once this form has been completed, an email is sent to the YAAM administrator and if the new data is verified it can be added to YAAM.

The *TUTORIAL* page offers two videos that show the user how to perform a *General search* and a *YAAM search*. Finally, the *LAST UPDATE* page contains information on the last time YAAM was updated. In this page, user can see the date when the last registry was added, the systematic name of the protein and the type of modification and residue.

## Results

YAAM describes the exact residue of the protein active site, the important residues to bind ions (Calcium and other metals) of 3089 residues and 12 PTMs (ubiquitination, phosphorylation, acetylation, lipidation, oxidation, succinylation, glycosylation, methylation, sumoylation, nitration, disulfide bond formation and N-terminal acetylation) for a total of 121 921 residues (Supplementary Table S2). All the information in YAAM is associated to an original source (database or published paper). A PTM can be included in the YAAM database only if the exact residue of such PTM was experimentally demonstrated by MS, point mutation or functional evidence.

YAAM database maps important PTM residues in a 3D canvas for about 680 proteins with a known 3D structure. YAAM can display 3D structures mapping each modification; all this information has been manually curated. The structures can be visualized in the most commonly used browsers without the need to install any plugin. The molecules are easy to visualize and manipulate even in mobile devices.

Additionally, each protein found in the database contains a brief description and its sub-cellular localization. In order to make the identification of the protein sub-cellular localization easier, we reduced the annotations from Gene Ontology Slim. We re-classified the GO annotation in 16 categories, based in the physiology of yeast. For example, during the yeast cell cycle the nucleus is never disassembled, for this reason all the proteins will be always in the nucleus like the components of the splicing machinery, the kinetochore and the spindle pole body. However, sometimes these components are not classified as nuclear proteins. It is important to remark that the information about protein localization is independent of the information on the PTMs. In the vast majority of cases, the PTMs are described without any reference of the protein localization and this was included in YAAM only for informational purpose.

## Example of YAAM search

**Figure 3.**
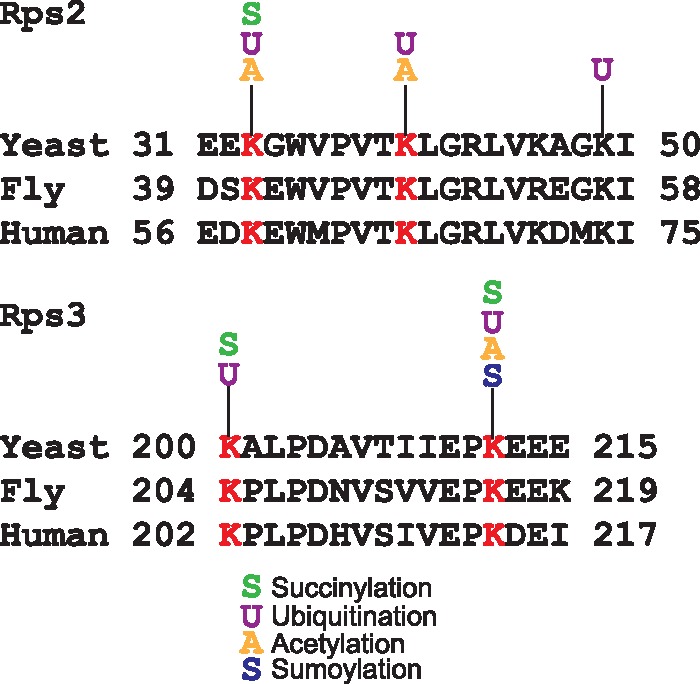
Alignments of Rps2 and Rps3 conserved ubiquitinated residues in yeast, fly and human. In red are shown the lysine residues reported as ubiquitinated in the three species. For each lysine the acetylated, succinylated, ubiquitinated or sumoylated residues experimentally reported in YAAM are shown.

Another remarkable feature of YAAM is that it offers the possibility to perform a visual analysis of certain modified regions of a protein sequence that could be involved in a regulatory mechanism, e.g. the coincidences of modifications in the same residue. For instance, lysine modifications are crucial residues for protein stability (5, 28), and when looking for coincidences in the same residue, YAAM helped to find proteins of which stability could be influenced by modifications in one residue. In order to demonstrate this, we performed an analysis for proteins that are acetylated, ubiquitilated and succinylated at the same residue. We found that translational elongation and cytoplasmic translation are the more enriched GO Terms for biological process (*P*-value of 4.868e–71 and *P*-value of 1.229e–64, respectively). On the other hand, the cytosolic ribosome is the cellular component with more coincidences of the three modifications at the same residue (*P*-value of 6.224e–73). Taking together these results suggest that protein translation could be regulated by these three PTMs. Even if the succinylation (29) and acetylation (30) of ribosomal proteins was described long time ago, the effects of these modifications on protein translation are not yet clear. The regulation of protein translation by these two PTMs seems to be an evolutionary conserved mechanism because there are coincidences of these modifications at the same residue in ribosomal proteins in bacteria, plants and vertebrates (16, 31–35). As regards ubiquitination, recently it was demonstrated that this modification plays an important role in translational regulation (36–38). Ubiquitination of the 40 S ribosome subunits regulates cell survival during chronic unfolded protein response activation (37). These ubiquitinations are conserved between yeast, fly and human. Furthermore, it was found that the levels of ubiquitination on K33 and K40 of Rps2 and K200 and K212 of Rps3 in yeast change after a treatment with a proteasome inhibitor (Figure 3). Interestingly, all these residues have at least two different PTMs reported. This suggests that the modification of these residues could be implicated on the ribosome regulation.

**Figure 4.**
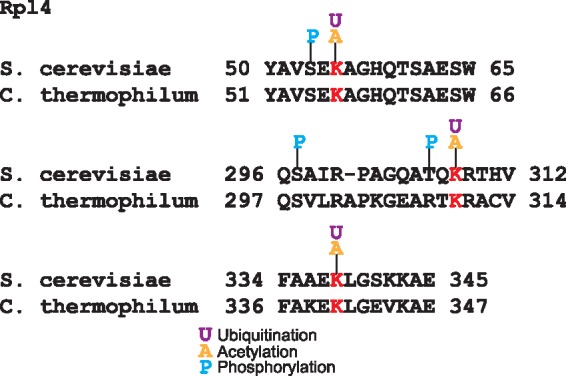
Alignment of Rpl4 ubiquitinated sites in *S. cerevisiae* and *C. thermophilum*. In red are the conserved lysine residues substrates of the ubiquitin ligase Tom1. These lysines are acetylated and ubiquitilated in yeast. In blue are shown the phosphorylated residues.

Other mechanism of ribosomal protein regulation is mediated by the interaction with chaperones that participate in ribosome biogenesis (39). The crystal structure of the complex of the ribosomal protein Rpl4 with the chaperone Acl4 from the fungus *Chaetomium thermophilum* showed that residues K56, K310 and K340 are in the Acl4 binding region (39). These three lysines are substrate of the ubiquitin ligase Tom1 (40); intriguingly these residues are conserved in yeast and they have been reported to be acetylated and ubiquitinated (Figure 4). Furthermore, two of these ubiquitination sites are near to phosphorylated residues; functional PTMs tend to be close to phosphorylation sites (4). This coincidence of PTMs over these residues suggests that both the acetylation and ubiquitination of these lysines are involved in the ribosome biogenesis regulation.

## Discussion

Although YAAM is a database that compiles information on PTMs in *S. cerevisiae*, it offers the possibility to visualize and find coincidences of PTMs in a single residue easily. With the information contained in YAAM the user can analyse spatially the PTMs what facilitates hypothesizing about the regulation of proteins using experimentally confirmed information of PTMs. We hope that YAAM will contribute to find molecular regulatory mechanisms of proteins that could be conserved in other species.

## Availability

YAAM database is freely available at http://yaam.ifc.unam.mx.

## Supplementary data

Supplementary data are available at *Database* Online.

## Supplementary Material

Supplementary Table 1

Supplementary Table 2
