# Supplementary material for: YAAM: Yeast Amino Acid Modifications Database
Source: Database (Oxford). 2018 Jan 9;2018:bax099. doi: 10.1093/database/bax099 (PMC7206644; doi:10.1093/database/bax099)
Supplement: Supplementary Table 1 [file bax099_supp_table_1.docx]

|  | Phosphorylation | Ubiquitylation | Acetylation | Sumoylation | Succinylation | Methylation | Last Update* |
| --- | --- | --- | --- | --- | --- | --- | --- |
| PTMCode | 13,912 | 2,372 | 3,123 | 43 | 3 | 138 | Sep-14 |
| dbPTM | 20,748 | 463 | 29 | 29 | 0 | 49 | Sep-12 |
| YAAM | 34,643 | 8,851 | 6,971 | 84 | 1,752 | 244 | Oct-17 |

Supplementary Table 1

Comparison of six classes of PTMs in three different databases. Each column contains the total number of modified residues for a determinate PTM in each one of the three databases compared.

*The last update corresponds to the date of the downloadable file.
