# Supplementary material for: YAAM: Yeast Amino Acid Modifications Database
Source: Database (Oxford). 2018 Jan 9;2018:bax099. doi: 10.1093/database/bax099 (PMC7206644; doi:10.1093/database/bax099)
Supplement: Supplementary Table 2 [file bax099_supp_table_2.docx]

| **Modification/important residue** | **Number of registries** |
| --- | --- |
| Acetylation | 10,035 |
| Active Site | 1,115 |
| Calcium binding site | 26 |
| Disulfide bond | 262 |
| Glycosylation | 1,970 |
| Lipidation | 181 |
| Metal binding site | 1,970 |
| Methylation | 283 |
| Nitration | 14 |
| N-terminal Acetylation | 758 |
| Oxidation | 869 |
| Phosphorylation | 87,703 |
| Succinylation | 1,752 |
| Sumoylation | 124 |
| Ubiquitynation | 14,880 |
| **Coverage** |  |
| Modified proteins | 4,896 |
| Percentage of modified proteins from the proteome | 72.89% |

Supplementary Table 2.

Number of entries for each PTM or important residues in YAAM up to October 2017. Each entry corresponds to a reported PTM, ion binding site or active site in a specific residue. A modified residue is entered each time that a PTM is described in literature, for this reason a PTM in a specific residue could be reported more than once.
